# 7,8-Dihydroxyflavone alleviates apoptosis and inflammation induced by retinal ischemia-reperfusion injury via activating TrkB/Akt/NF-kB signaling pathway

**DOI:** 10.7150/ijms.65733

**Published:** 2022-01-01

**Authors:** Aihua Yu, Shun Wang, Yiqiao Xing, Mengyao Han, Kun Shao

**Affiliations:** 1 Eye Center, Renmin Hospital of Wuhan University, Wuhan University, Wuhan 430060, Hubei Province, China; 2 Department of Ophthalmology, Zhongnan Hospital of Wuhan University, Wuhan University,Wuhan 430071, Hubei Province, China

**Keywords:** Retinal ischemia-reperfusion injury, 7,8-dihydroxyfavone, BDNF, TrkB, Apoptosis.

## Abstract

Retinal ischemia-reperfusion injury (RIRI) is of common occurrence in retinal and optic nerve diseases. The BDNF/TrkB signaling pathway has been examined to be neuroprotective in RIRI. In this study, we investigated the role of a potent selective TrkB agonist 7,8-dihydroxyfavone (DHF) in rat retinas with RIRI. Our results showed that RIRI inhibited the conversion of BDNF precursor (proBDNF) to mature BDNF (mBDNF) and increased the level of neuronal cell apoptosis. Compared with RIRI, DHF+RIRI reduced proBDNF level and at the same time increased mBDNF level. Moreover, DHF administration effectively activated TrkB signaling and and downstream Akt and Erk signaling pathways which increased nerve cell survival. The combined effects of mBDNF/proBDNF increase and TrkB signaling activation lead to reduction of apoptosis level and protection of retinas with RIRI. Moreover, it was also found that astrocytes labeled by GFAP were activated in RIRI and NF-kB mediated the increased expressions of inflammatory factors and these effects were partially reversed by DHF administration. Besides, we also used RNA sequencing to analyze the differently expressed genes (DEGs) and their enriched (Kyoto Encyclopedia of Genes and Genomes) KEGG pathways between Sham, RIRI, and DHF+RIRI. It was found that 1543 DEGs were differently expressed in RIRI and 619 DEGs were reversed in DHF+RIRI. The reversed DEGs were typically enriched in PI3K-Akt signaling pathway, Jak-STAT signaling pathway, NF-kB signaling pathway, and Apoptosis. To sum up, the DHF administration alleviated apoptosis and inflammation induced by RIRI via activating TrkB signaling pathway and may serve as a promising drug candidate for RIRI related ophthalmopathy.

## Introduction

Retinal ischemia-reperfusion injury (RIRI) is the recovery of perfusion after acute ischemia of the retina, causing irreversible structural and metabolic changes in the retinal tissue 1. It is easy to damage the retinal nerve tissue and cause varying degrees of visual function impairment. Acute retinal ischemia is clinically common in acute high intraocular pressure, retinal vascular embolism disease and intraocular surgery 2.

At present, the mechanism of RIRI mainly includes various theories such as apoptosis, increase of free radicals and inflammatory factors, and decrease of neurotrophic factors 3. Brain derived neurotrophic factor (BDNF) is a protein synthesized by nerve cells and is mainly distributed in the central nervous system and peripheral nervous system. The main function of BDNF is to affect the development, growth and differentiation of retinal neuron cells during the embryonic period, and to have a certain impact on the survival of differentiated ganglion cells 4. Current studies have shown that when culturing retinal nerve cells *in vitro*, BDNF can increase the number of nuclei and the thickness of the plexiform layer, and at the same time promote the formation and maturation of synapses by regulating the bifurcation of dendrites and axons 5,6. In the retinal ischemia-reperfusion model, BDNF plays a very important role in the functional recovery of experience-dependent retinal cells 7. In animal models of retinal ischemia-reperfusion, early injection of BDNF into the vitreous cavity can reduce the apoptosis of retinal nerve cells. The Schwann cells transfected with BDNF gene were injected into the vitreous cavity to elevate BDNF expression in the retina, and it was found that BDNF has the effect of nourishing retinal nerve cells 8. The post-translation processing of BDNF happened in the nervous system, that is, precursor of BDNF (proBDNF) is converted into mature BDNF (mBDNF) by shearing action of calcium-dependent protease Furin 9. ProBNDF and mBDNF have completely different physiological functions. ProBDNF can induce the apoptosis of nerve cells by activating p75NTR signaling pathway, while mBDNF can promote the formation of axons and dendrites of nerve cells and delay the apoptosis of nerve cells through activating TrkB signaling pathway 10,11.

Due to the protective roles of BDNF in nerve cells, any changes involved in the production, maturation and signal transduction pathways of BDNF will cause damage to the optic nerve. 7,8-Dihydroxyflavone (DHF) is a potent mimetic material of BDNF and has been exhibited neuroprotective properties in several retinal injury models 12-14. However, there is still lacking evidences to support its neuroprotective roles in RIRI. In this study, RIRI models of rats were established and the pathological changes were observed in the retina tissue. Moreover, DHF was used to treat RIRI rats and the responses of DHF administration in the injured retina were detected by RNA-seq analysis. We want to investigate whether DHF can exhibit neuroprotective functions in RIRI and the related mechanism of its action.

## Materials and methods

### Animals

Healthy male Sprague-Dawley (SD rats), 7-week-old and weighted 200-220 g, were supplied by the Animal Experimental Center of Zhongnan Hospital, Wuhan University, and housed in SPF grade animal feeding rooms (temperature 20-25℃, humidity 40%) under a 12 h-12 h of light to dark cycle condition. All the animal experiments were ratified by the Animal Experiment Center and Ethics Committee of Zhongnan Hospital of Wuhan University and also followed the National Institutes of Health Guide for the Care and Use of Laboratory Animals. There was no obvious abnormality for all animals in ocular examination and general condition. General condition requirements: hair with color bright, limbs without obvious fracture or scar and disease signs. Eye examination: the eyelids of both eyes are complete, the cornea was bright, the anterior chamber was visible, the pupil was round without obvious abnormal pupil shape, and the iris was white. All rats were routinely fed for 7 days before the experiment to adapt to the environment.

### Rat model of retinal ischemia-reperfusion injury

**Figure 1:**
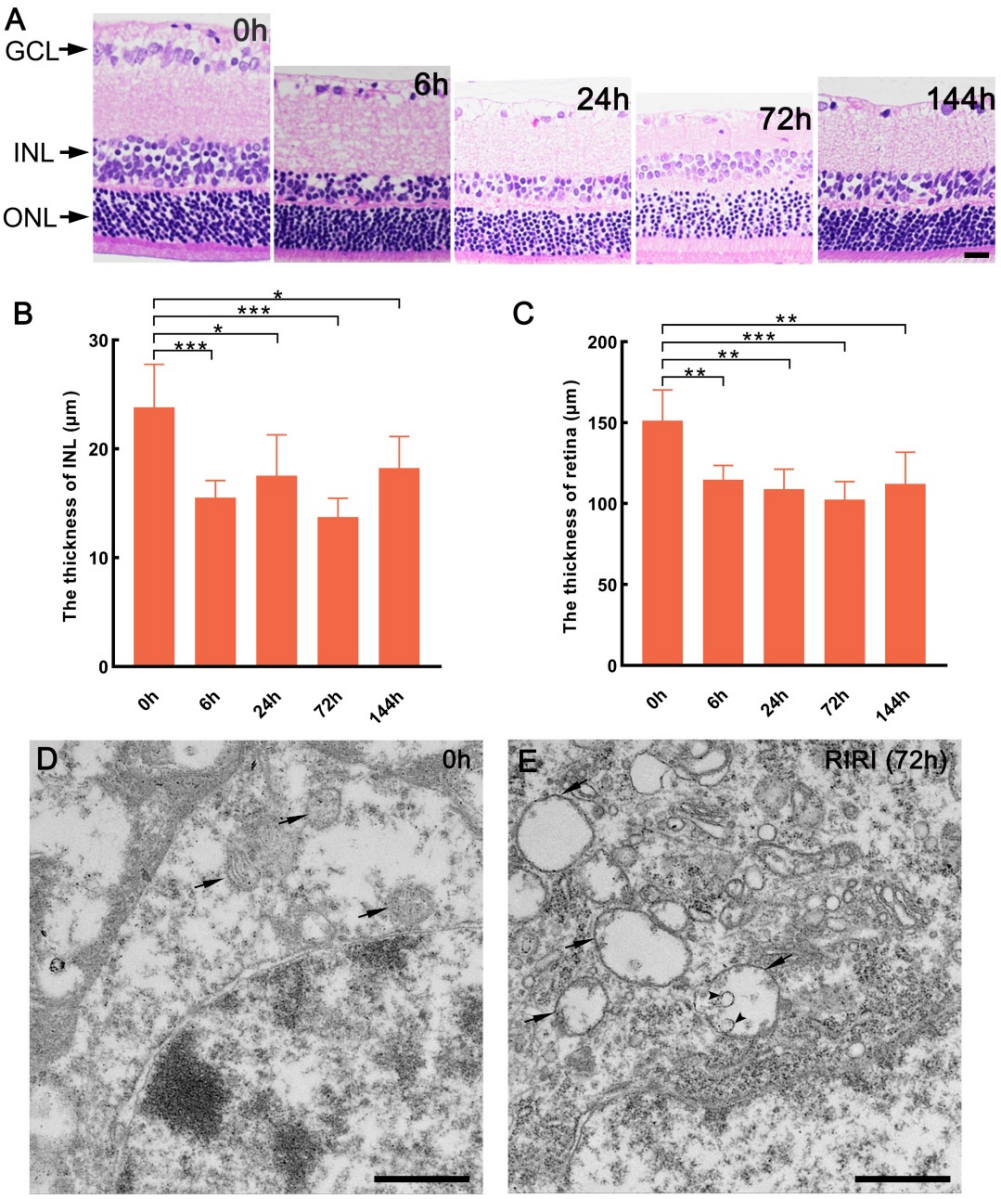
Histologic and mitochondrial changes of the retina induced by RIRI in rats. (A) Hematoxylin and eosin staining of retina for 0 h and different reperfusion time group. (B and C) The statistical data on thickness of inner nuclear layer (INL), and the whole retina. Scale bar: 20 μm (A). (D and E) Mitochondrial structure observation of 0 h and RIRI after 72 h. Arrows show the mitochondria and the arrow heads show the shedding inner membrane in mitochondria. GCL: ganglion cell layer. ONL: outer nuclear layer. ***p < 0.001, **P < 0.01, *P < 0.05, ANOVA test was used for comparing the values between contol (0 h) and RIRI series (6 h, 24 h, 72 h, and 144 h) (n = 5 determinations for each). Scale bars: 1 μm (D and E).

Before the experiment, the animals were weighted and narcotized by abdominal cavity injection with 10% (w : v) chloral hydrate (3 mL/kg), then 3 drops of levofloxacin were dropped on to the eyes, and the right eye was anesthetized to dilate the pupils with tropicamide and prometvacaine hydrochloride. Ofloxacin ophthalmic gel was used to cover the cornea of the rats after anterior chamber puncture in the left eye, which was used as the Sham group. A 30-gauge sterile needle connected to a sterile plastic infusion tube was pricked into the anterior chamber of right eye horizontally, and the position of the needle was fixed. Then a normal saline bag attached to the other end of the infusion tube was raised to a height of 150 cm, which can make the intraocular pressure increase to about 110 mmHg in the eyes. The needle was removed gradually until the ischemic condition was kept for 60 min. The conjunctival congestion could be seen after the needle was pulled out, and the retina of the rats changed from white to orange, indicating that the retinal blood vessels had resumed perfusion. Propivacaine hydrochloride and levofloxacin were continuously dropped on to the eyes during the maintenance of high pressure perfusion. After needle removal, ofloxacin gel was applied to cover the conjunctiva of the rat to prevent infection. Animals were killed respectively at 0 hours, 6 hours, 24 hours, 72 hours, and 144 hours after RIRI, retinas or eyeballs were collected for the following analysis in Figure 1.

### Drug Administration

**Figure 2:**
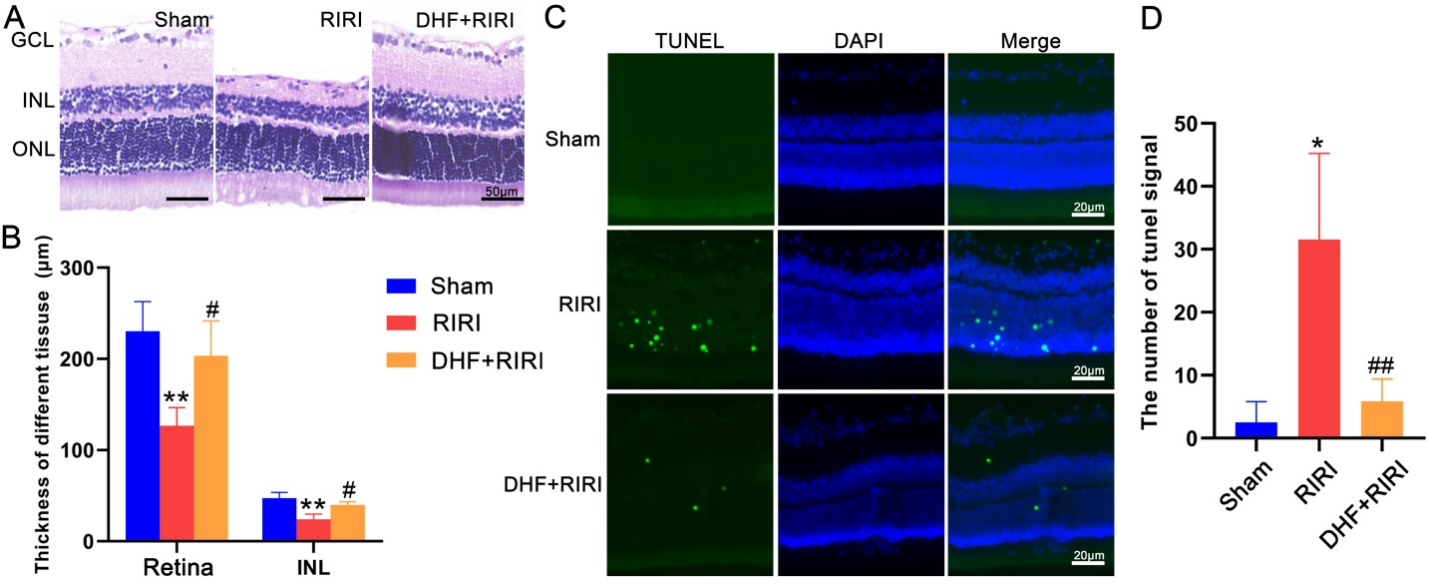
The effect of 7, 8-Dihydroxyflavone on RIRI in rat. (A) Hematoxylin and eosin staining of retina for Sham, RIRI, and DHF+RIRI groups. (B) The statistic of thickness of inner nuclear layer (INL) and the whole retina in different groups. (C and D) TUNEL test and statistic of TUNEL signal in Sham, RIRI, and DHF+RIRI samples. GCL: ganglion cell layer. ONL: outer nuclear layer. **P < 0.01, *P < 0.05 for RIRI compared with Sham, ##P < 0.01, #P < 0.05 for DHF+RIRI compared with RIRI, by T-test (n = 5 determinations for each). Scale bar: 50 μm (A), 20 μm (C).

**Figure 3:**
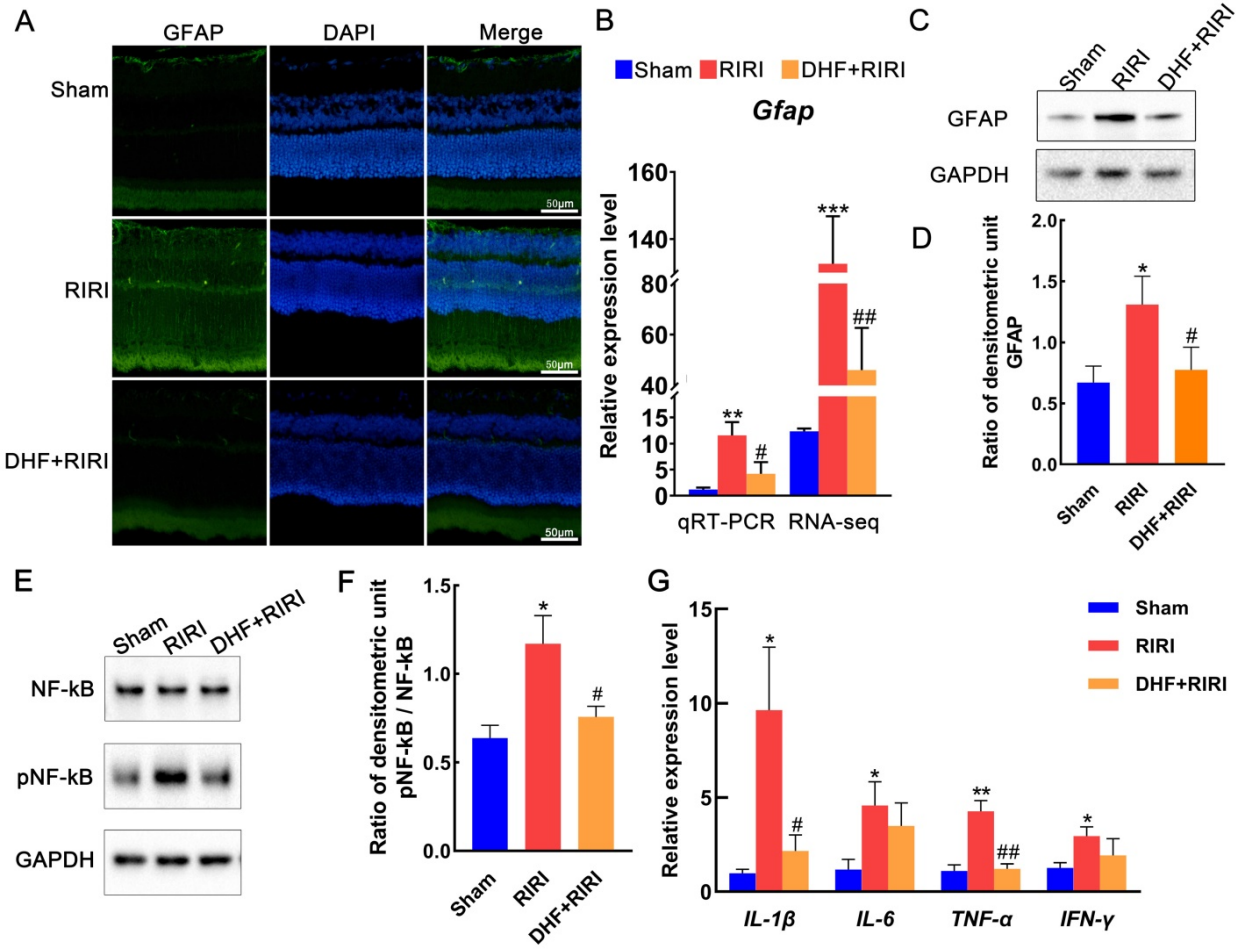
The alterations of GFAP expression in RIRI and DHF+RIRI. (A) Immunofluorescence analysis of anti-GFAP in Sham, RIRI and DHF+RIRI. (B) qRT-PCR analysis and RNA-seq data for GFAP in different conditions. (C and D) Western blot analysis and statistics of GFAP in different conditions. (E and F) Western blot analysis and statistics on pNF-kB and NF-kB. (G) qRT-PCR analyses for *IL-1β*, *IL-6*, *TNF-α*, and *IFN-γ*. ***P < 0.001, **P < 0.01, *P < 0.05 for RIRI compared with Sham, ##P < 0.01, #P < 0.05 for DHF+RIRI compared with RIRI, by T-test (n = 3 determinations for each). Scale bar: 50 μm.

**Figure 4:**
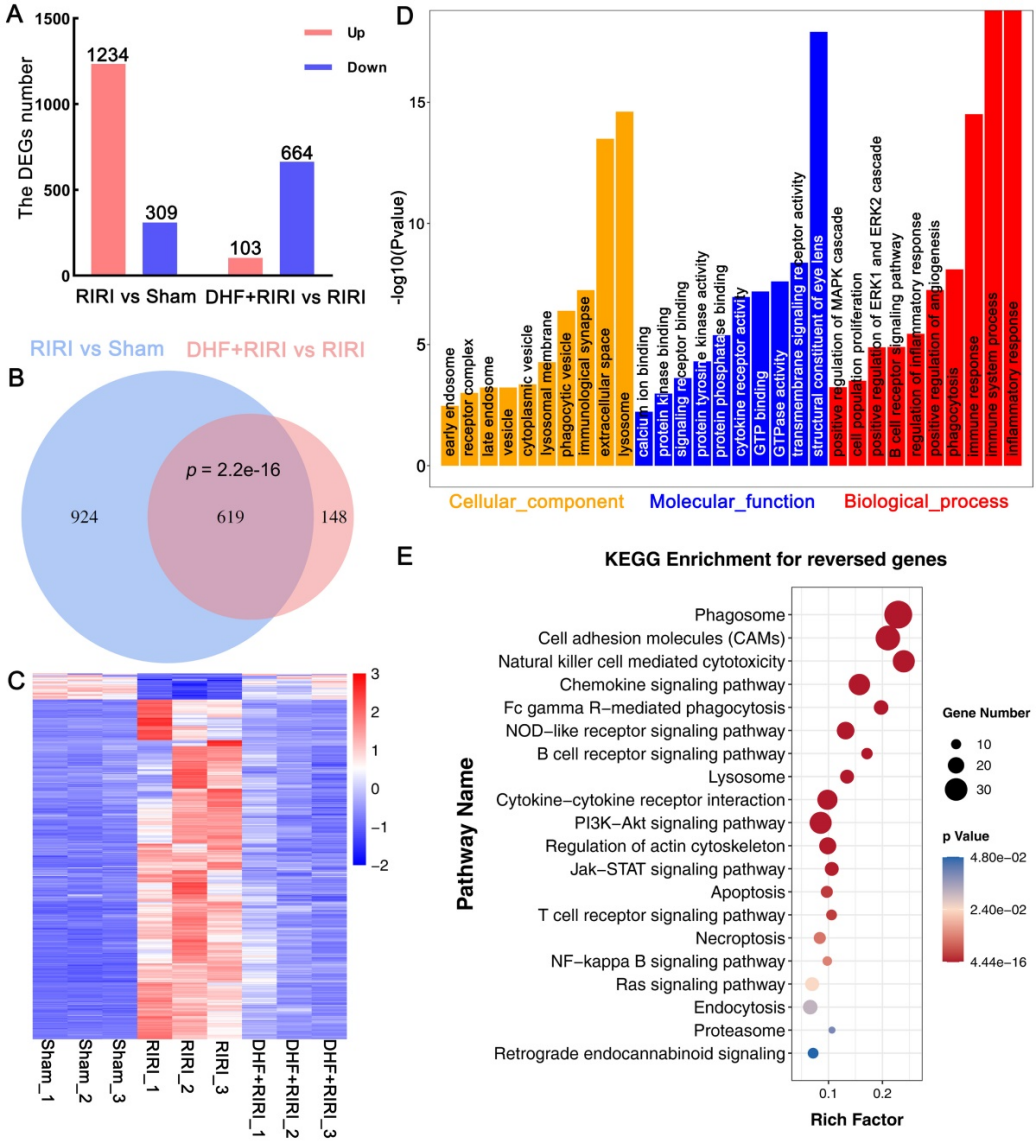
RNA-seq analysis on Sham, RIRI, and DHF+RIRI in rat. (A) Histogram of deferentially expressed genes (DEGs) between different groups. (B) Venny analysis between DEGs in RIRI vs Sham and DEGs in DHF+RIRI vs RIRI. (C) Expression heatmap of 619 reversed genes. (D and E) GO enrichment analysis and KEGG enrichment analysis based on the reversed genes.

**Figure 5:**
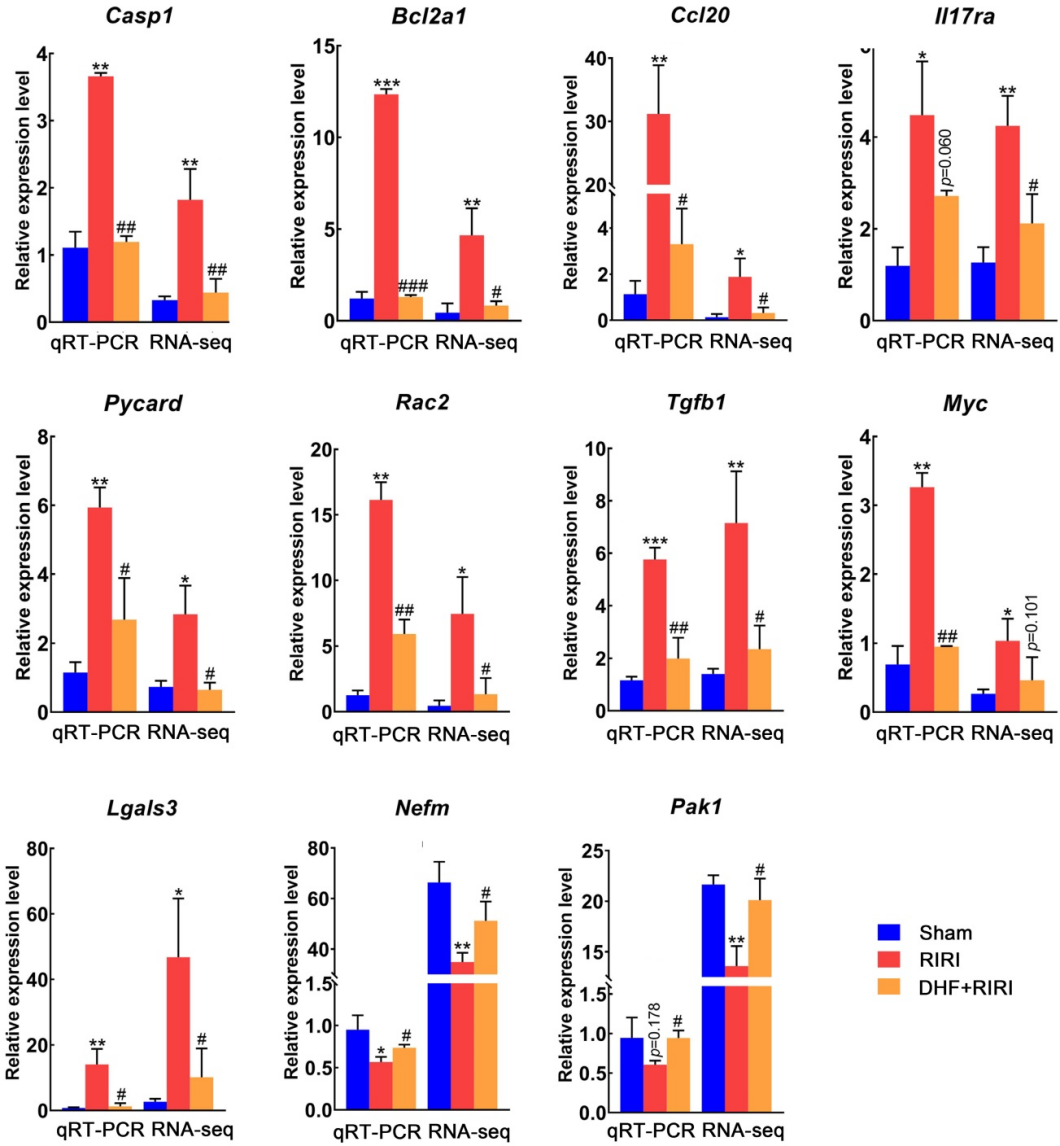
Expression validation of differently expressed genes by qRT-PCR. ***P < 0.001, **P < 0.01, *P < 0.05 for RIRI compared with Sham, ###P < 0.001, ##P < 0.01, #P < 0.05 for DHF+RIRI compared with RIRI, by T-test (n = 3 determinations for each).

**Figure 6:**
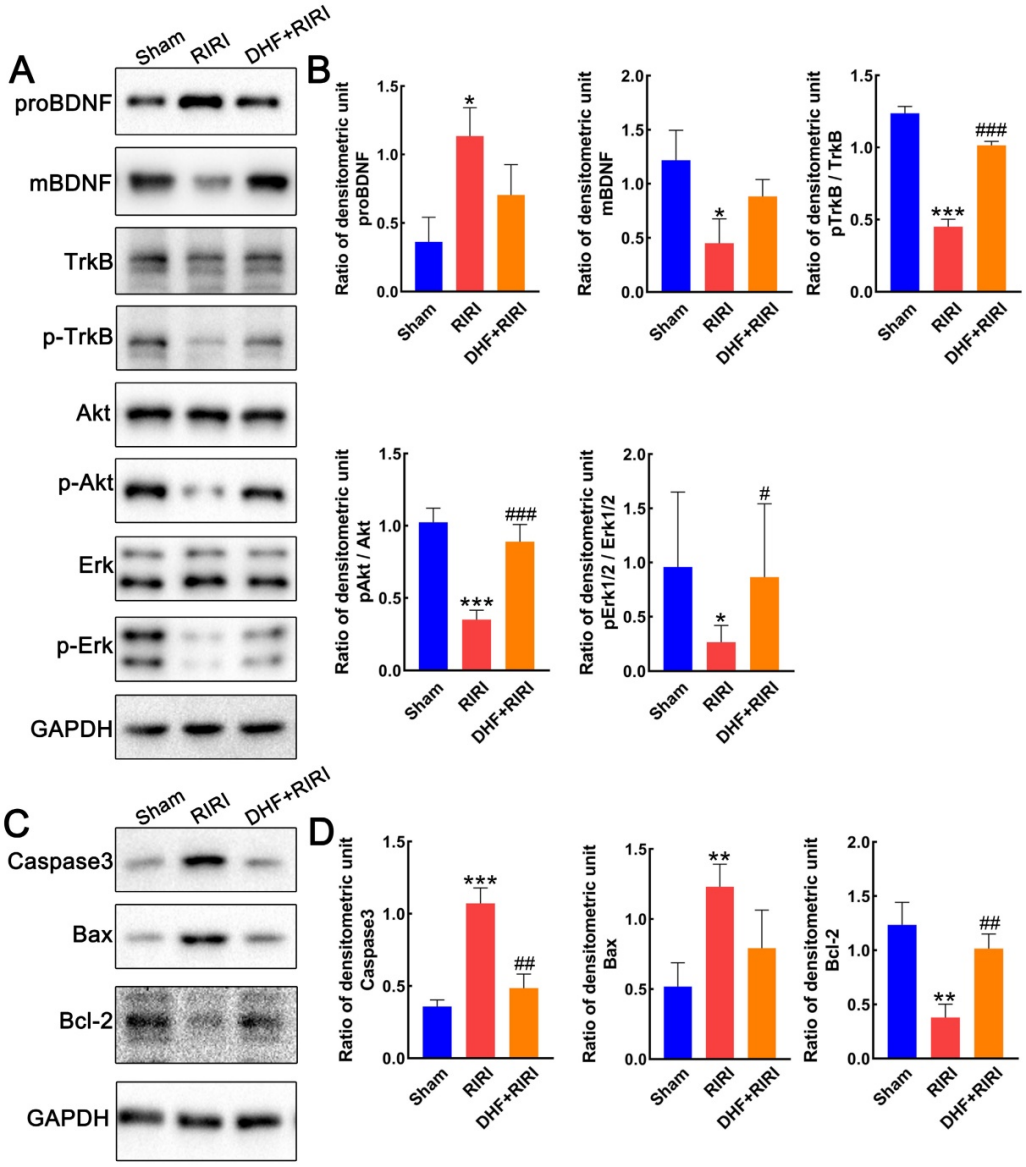
Expression alteration of BDNF/TrkB and apoptosis pathway. (A and B) Western blot analysis and statistics on proBDNF, mBDNF, TrkB, pTrkB, Erk1/2, pErk1/2. (C and D) Western blot analysis and statistics on Caspase3, Bax, and Bcl-2. ***P < 0.001, **P < 0.01, *P < 0.05 for RIRI compared with Sham, ###P < 0.001, ##P < 0.01, #P < 0.05 for DHF+RIRI compared with RIRI, by T-test (n = 3 determinations for each).

For the Sham group, ischemia-reperfusion was not done and the solvent (10% DMSO and 90% corn oil) was applied by intraperitoneal injection. In the RIRI group, ischemia-reperfusion was induced and then the solvent was also injected by intraperitoneal injection. For 7,8-Dihydroxyflavone (DHF)+RIRI group, DHF (dissolved in 10% DMSO and 90% corn oil), 10 mg/kg (MCE, USA), was injected intraperitoneally after completion of the ischemic condition. Six days (144 hours) later, retinas or eyeballs were collected for the following analysis in Figure 2-6.

### Hematoxylin and eosin staining and quantification of the retinal thickness

After the RIRI model was constructed, eyes were enucleated from anesthetized rats. The conjunctiva, the muscle and connective tissue outside the eye were removed. After removing extraocular tissues, the samples were fixed in 4% paraformaldehyde for 2 hours, then fixed in 4% neutral paraformaldehyde, embedded in paraffin and sectioned. Slides were stained by hematoxylin solution for 90 seconds followed by another water wash for 15 min. Then, slides were stained by eosin solution for 30 seconds, and dehydrated by ethanol. Mounting medium was applied once slides were dried and the slides were cover-slipped and photographed under a light microscope. Five points of retinal or inner nuclear layer (INL) thickness were measured in a section at least by Digimizer (https://www.digimizer.com/index.php), and evaluated the averages of them as the value of an eye. All the data were calculated and showed by GraphPad Prism 8.

### Transmission electron microscope for mitochondria

Fresh retina tissues with total volume less than 1 cm^3^ were fixed for 2-4 h in 3% glutaraldehyde under 4 ℃, then washed 3 times with 0.1 M PBS buffer (pH=7.4) and 15 minutes for each. 1 % osmic acid was used for fixing until 2 hours under room temperature, after that washed 3 times with 0.1 M PBS. The samples will be dehydrated with the gradient alcohol, such as 50%-70%-80%-90%-95%-100%-100%, and 15 minutes for each. Then the samples will be incubated with the solution (acetone : spon812 = 1 : 1) over night, and polymerized for 48 hours under 60 ℃. The samples for ultrathin section (60-70 nm) were stained with uranyl acetate and lead citrate, and then were observed and photographed under a transmission electronic microscope (JEOL JEM-1400plus, Japan).

### Western blot assay

After isolation of retina, the tissue samples were grinded into fine powder in liquid nitrogen with a mortar and pestle. The cell lysis buffer for Western and IP (Beyotime Biotech, China), mixed with protease and phosphatase inhibitor cocktail (50X, P1050, Beyotime Biotech, China) for mammalian cell and tissue extracts, was applied to the powder for cell lysis (one hour on the ice). Protein concentrations were measured using NanoPhotometer N60. 50 μg protein sample per lane was separated on 10% SDS-PAGE and then was transferred onto a PVDF membranes (Millipore, USA). The membranes were blocked in 5% skim milk in Tris-buffered saline with 0.5% Tween 20 (TBST) for 1-2 hours at room temperature on a shaking table. The membrane was incubated overnight at 4 ℃ with primary the antibodies, including mBDNF (ABclone, 1 : 1000, China), proBDNF (Santa, 1 : 500, USA), TrkB (CST, 1 : 1000), pTrkB (CST, 1: 1000, USA), Akt (CST, 1: 1000), pAkt (CST, 1: 1000), Erk1/2 (CST, 1: 1000), pErk1/2 (CST, 1: 1000) Caspase3 (ABclone, 1 : 1000), Bax (ABclone, 1 : 1000), Bcl-2 (CST, 1: 1000), GFAP (ABclone, 1 : 1000), NF-kB (ABclone, 1 : 1000), Phospho-NF-kB (ABclone, 1 : 1000) and GAPDH (ABclone, 1 : 1000). On the next day, after washing with TBST by 3 times and 5 min for each, membranes were incubated with horseradish peroxidaseconjugated secondary antibody (1 : 5000, Beyotime Biotech, China) on the shaking table for 2 hours at room temperature. The membranes will be washed with TBST, and after that, protein bands were detected by using ECL reagent (Vazyme Biotech Co.,Ltd, China) and obtained images after exposure on Bio-Rad ChemiDoc XRS^+^. With the help of Image Lab, an image analysis software, the intensity of the protein band was semiquantitatively measured. GAPDH was used for internal reference.

### Immunofluorescence assay

An *In situ* Cell Apoptosis Detection Kit (Tunel assay, Boster,Wuhan, China) was used to monitor the apoptotic positive cells on the paraffin-embedded retina sections. We chose five different areas in each retinal section to count the Tunel-positive cells. The positive cell numbers per unit length of retinal surface in the 5 areas were reported. Three samples per group were used in this assay.

Paraffin section of retina was incubated under 65 ℃ for 2 h, and then washed with PBS (0.01M) by 3 times after dewaxing and 5 min for each. Antigen retrieval was finished with EDTA repair liquid and then washed with PBS by 3 times. 3% peroxide was used to incubate for the samples for 10 min in dark. The sections were incubated overnight at 4 ℃ with anti-GFAP (ABclone, 1 : 200), and after washing with PBS, they were incubated with secondary antibody for 1 h. Finally, the sections were incubated with DAPI before covered with coverslip and observed under fluorescence microscope. The average flourescence intensity was calculated by Image J (v1.46r).

### RNA-seq analysis and qRT-PCR confirmation

Total RNA was extracted using TRIzol reagent (Invitrogen, Carlsbad, CA, USA) following the manufacturer's procedure. 5 μg total RNA was used to purify the poly(A) RNA by using Dynabeads Oligo (dT)25-61005 (Thermo Fisher, CA, USA). Then the poly(A) RNA was used for construction of cDNA library. At last, 2×150 bp paired-end sequencing (PE150) was performed on an Illumina Novaseq™ 6000 (LC-Bio Technology CO., Ltd., China) following the vendor's recommended protocol.

Cutadapt software was used to get rid of the reads that contained adaptor contamination. HISAT2 software was used to map the reads to the rat genome (ftp://ftp.ensembl.org/pub/release-101/fasta/rattus_norvegicus/dna/). Then, StringTie was used to assemble the mapped reads came from each sample with default parameters. And, all transcriptomes were merged to reconstruct a comprehensive transcriptome using gffcompare software. After that, StringTie and ballgown were used to estimate the expression levels and perform expression level for mRNAs by calculating FPKM. The differentially expressed mRNAs were screened with |log2(fold change)| > 0.5 and p value < 0.01 by R package edgeR, and then were used for GO enrichment and KEGG enrichment analysis. The heatmap, venny analysis, GO and KEGG enrichment analysis were performed by OmicStudio (https://www.omicstudio.cn/).

qRT-PCR was performed according to a previous study 15 and primers used in this study were showed in supplemental file Table S3.

## Results

### Retina changed apparently after retinal ischemic-reperfusion injury in rat

To gain insight into the characterization of retinal ischemic-reperfusion injury (RIRI), we established an *in vivo* rat model under different reperfusion time and observed the tissue structure integrity by H&E staining. The result showed that the cell morphology and volume of ganglion cell layer (GCL) had apparent changes with different reperfusion time (Figure 1A). At the same time, the inner nuclear layer (INL) was thinner in the RIRI group compared with 0 h group, the cells were arranged sparsely, especially at 6 h after ischemic-reperfusion injury (Figure 1A and B). Moreover, the cells of outer nuclear layer (ONL) more sparsely arranged as well after 24 and 72 hours ischemic-reperfusion (Figure 1A). In addition, we also found that the whole retina was thinner after RIRI occurred than before (Figure 1A and C). As a result, RIRI can obviously change the tissue structure of retina in rats, and it's meaningful to make clear about how this process was regulated.

The structure of mitochondria was also observed in our study. The result showed that in 0 h group, the mitochondria maintained intact structure including abundant mitochondrial matrix and cristae (Figure 1D), however, we found many vacuolar mitochondria with very few matrix in the RIRI group (72 h) (Figure 1E). Sometimes, in the abnormal mitochondria, some circled structure, formed by inner membrane shedding from mitochondrial outer membrane, will be observed (Figure 1E). As a result, RIRI can disrupt mitochondrial membrane structure in rat retina.

### 7, 8-Dihydroxyflavone protected the retina after RIRI

In order to confirm the role of BDNF in RIRI response, a selective receptor agonist for TrkB, 7, 8-Dihydroxyflavone (DHF), was used for RIRI. The results of HE staining (Figure 2A) and statistical analysis (Figure 2B) showed that the thickness of INL and the whole retina were thinner in RIRI group than those in Sham group. However, in DHF+RIRI group, the thickness of INL and retina were almost the same as the Sham group (Figure 2A and B). We also did TUNEL assay, and found that the increased TUNEL signal in RIRI was reduced after DHF treatment (Figure 2C and D).

Glial fibrillary acidic protein (GFAP) is a marker for activated astrocytes 16. Activated glial cells expressing increased GFAP levels were detected in glaucoma animal models 17 and human glaucomatous donor eyes 18. In the RIRI group, the GFAP signals were increased compared with Sham. But after DHF treatment, GFAP expression reduced to approximately the same as Sham (Figure 3A). The results of qRT-PCR (Figure 3B) and western blot (Figure 3C and D) also confirmed these findings. NF-kB and phosphorylated NF-kB (pNF-kB) were also tested by western blot in our research. And the results showed that pNF-kB/NF-kB was significantly increased in RIRI and almost recovered by DHF (Figure 3E and F). At mRNA level, we also found that some inflammatory factors, such as IL-1β, IL-6, TNF-α, and IFN-γ were all induced by RIRI and restored after DHF treatment (Figure 3G).

### Expression profile analysis of rat retinas in RIRI and DHF+RIRI

To explore the molecular mechanism in rat RIRI, RNA-seq analysis was carried out based on Sham, RIRI and DHF+RIRI samples. Through whole transcriptome profiling, we identified a cascade of genes and pathways potentially involved in DHF effects. 1234 deferentially expressed genes (DEGs) were up-regulated and 309 DEGs were down-regulated in RIRI compared with Sham (Figure 4A and Table S1), and 103 DEGs were up-regulated and 664 genes were down-regulated due to the effect of DHF (Figure 4A and Table S1) (|log2FC| > 0.5 and p < 0.01). Among the 1543 DEGs in RIRI, there were 619 DEGs were reversed by DHF administration, including 575 up-regulated genes and 44 down-regulated genes (Figure 4B and Table S1). The heat map and clustering analysis based on the expression of 619 genes was also performed (Figure 4C). These results suggested that the DHF may reverse the expression of these genes and these genes may play essential role in RIRI response.

To further understand the function of these genes, Gene Ontology (GO) and Kyoto Encyclopedia of Genes and Genomes (KEGG) analysis were done. The 619 DEGs reversed by DHF were enriched in inflammatory response, immune system process, immune response, phagocytosis, positive regulation of angiogenesis, positive regulation of ERK1 and ERK2 cascade, lysosome and some endosome related GO term (Figure 4D and Table S2). These genes were also enriched in phagosome, cell adhesion molecules (CAMs), lysosome, apoptosis, necroptosis, PI3K-Akt signaling pathway, Jak-STAT signaling pathway, and some immune-related pathways (Figure 4E and Table S2). These results suggested that DHF may alleviate RIRI by affected the inflammatory response, immune response, and the BDNF/TrkB signaling pathway.

To make sure about the RNA-seq result, the expression levels of a set of genes, selected from the reversed DEGs, were assayed by quantitative real-time PCR (qRT-PCR). It was found that 9 genes (*Casp1*, *Bcl2a1*, *Ccl20*, *Il17ra*, *Pycard*, *Rac2*, *Tgfb1*, *Myc*, and *Lgals3*) were significantly up-regulated in RIRI, and 8 of them were significantly down-regulated in DHF+RIRI and the expression level of 7 DEGs was almost recovered with the same as Sham (Figure 5). Moreover, there were 2 genes (*Nefm* and *Pak1*) down-regulated in RIRI and reversed by DHF (Figure 5). These results proved the RNA-seq analysis was credible.

### BDNF/TrkB signaling pathway is activated in RIRI after DHF administration

To make clear about the role of BDNF-TrkB signaling in RIRI regulation, the expression levels of BDNF and TrkB in retina after RIRI were firstly tested by western blot. Interestingly, the precursor form of brain-derived neurotrophic factor (proBDNF) was increased after RIRI but reduced after DHF treatment, while the mature BDNF showed reverse expression pattern (Figure 6A and B). As a ligand for mature BDNF, the TrkB did not have any expression changes, however, the expression of phosphorylated TrkB (pTrkB) was lower in RIRI than Sham and restored by DHF treatment (Figure 6A and B). The downstream factors, pAkt/Akt and pErk/Erk, were also monitored in different groups in our study. They all inhibited by RIRI and can be restored by DHF (Figure 6A and B). Caspase 3 and Bax increased in RIRI but restored by DHF, however Bcl-2 was inhibited in RIRI and also restored by DHF (Figure 6C and D). These results suggested that DHF activated BDNF/TrkB signaling pathway and reduced the level of apoptosis induced by RIRI.

### Potential relationship between Furin and BDNF

**Figure 7:**
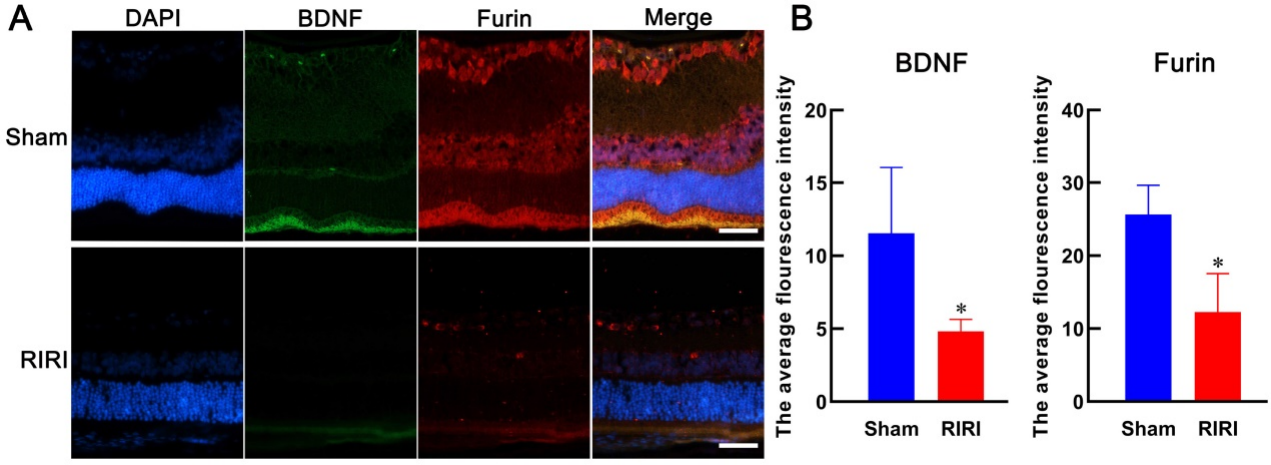
Expressions of Furin and BDNF in retinas of rat. Anti-BDNF (green stained) and anti-Furin (red stained) were used to detect their expression levels in retina by immunofluorescence. Both of them were deduced after RIRI. The average flourescence intensity was calculated by Image J and the histogram was made by GraphPad Prism 8. *P < 0.05 for RIRI compared with Sham by T-test (n = 3 determinations for each). Bars = 50 μm.

Furin maybe an important regulator of BDNF (30-33). To validate this, we detected their expression level in retina by immunofluorescence. The results showed that Furin and BDNF colocalized in most areas in retina and both of them were deduced after RIRI (Figure 7). These results suggested that the expressions of Furin and BDNF were positively correlated and Furin might execute the role of processing proBDNF to mBDNF.

## Discussion

Retinal ischemia-reperfusion injury (RIRI) is a common pathological change in ophthalmology and many ophthalmic diseases with high intraocular pressure cause damages to visual function through several mechanisms include inflammatory cascade, free radicals and calcium overload 19. In this study, we investigate the neuroprotective effects of DHF on retinal and optic neurons in RIRI rat models for the first time. We confirmed that RIRI could increase apoptosis and inflammation level in the retinas of rat. DHF inhibit astrocyte activation, inflammation and apoptosis level in RIRI by activating TrkB/Akt/NF-kB signaling and regulating Bcl-2/Bax apoptosis signaling.

As a nerve growth factor, BDNF plays an important role in the growth and development of nerve cells and the maintenance of nerve cell survival. A series of studies have documented that the decrease of BDNF in protecting neuronal survival following diverse types of retinal injuries 7-8. It is reasonable to believe that increasing BDNF levels can improve nerve cell survival based on not only restoring normal BDNF levels in retina tissue to maintain nerve cell vitality but also using more BDNF to stimulate nerve cell growth. Compared with BDNF injection or expression, the DHF (a mimetic flavonoid of BDNF) administration has its advantages in crossing the blood brain barrier when administered systemically and inducing TrkB response more quickly, enduringly, and robustly 20. Despite *in vitro* studies suggest protective effects of DHF against excitotoxic and oxidative stress on RGC lines and high glucose induced apoptosis on retinal pigment epithelial (RPE) cell 21, few studies have explored the neuroprotective effects of DHF in the stressed retina. We have examined that DHF administration ameliorates apoptosis level of retina induced by RIRI. Similar to our findings, recent studies have shown that DHF protects immature retina against hypoxic-ischemic injury 12, protects retinal ganglion cells against chronic intermittent hypoxia 13 and intraorbital optic nerve transection 14, and inhibits optic nerve degeneration in Wolfram syndrome rat model 22.

The retina is a component of the central nervous system (CNS) and its stress response is like that of CNS. Pathological conditions such as ischemia, neurodegeneration and inflammation lead to activation of astrocytes are associated with an up-regulation of the intermediate filament GFAP 16-18. Previous reports indicated that RIRI led to a higher expression level and immune mobility of GFAP 23,24. We also found that astrocytes were activated by detecting GFAP immunofluorescence in RIRI retina and the GFAP mRNA was also up-regulated in RNA-seq analysis and qRT-PCR confirmation. Moreover, DHF can inhibit the expression of GFAP, indicating that DHF can reduce reactivation of astrocytes after RIRI. We found the NF-kB mediated inflammatory signaling pathway was activated in RIRI reversed by DHF treatment. Previous reports indicated that the activated astrocytes secreted inflammatory factors such as TNF-α, IL-1β, IL-6, IFNγ, and COX-2, which in turn further promoted astrocyte activation, forming a vicious cycle 25. We speculated that DHF might inhibit the activity of astrocytes through Akt/NF-kB signaling and reduced the levels of inflammatory factors to protect the nerve cells in ischemic retina like previous report about the roles of RNase on RIRI 24. However, the precise mechanism on DHF and reactivation of astrocytes still needs to be explored in further study.

Previous studies have shown that DHF has neuroprotective effects with increasing nerve cell survival neurotrophic activities in psychiatric diseases by mediating MAPK/Erk, PI3K/Akt, and PLCγ signaling pathways following TrkB activation 26, 27. DHF could have a similar effect in TrkB signaling which agrees with our present findings. Our data showed that DHF promoted TrkB phosphorylation and up-regulated the expression of BDNF, and increased the Erk phosphorylation and Akt phosphorylation level. However, DHF protected RIRI retina via not only TrkB signaling pathway but also other mechanisms such as antioxidant and antiinflammatory effects 28. Moreover, our results showed that RIRI increased the BDNF precursor (proBDNF) level and decreased the mature BDNF (mBDNF) level, which increased neuronal cell apoptosis. ProBDNF can mediate the apoptosis of nerve cells after binding to the p75NTR receptor 29. Interestingly, the proBDNF and mBDNF levels were restored to normal conditions after systematic DHF administration. It was speculated that the function of proprotein convertases like Furin that activates the precursor protein into a biologically active form was returned to normal in RIRI retina under systematic DHF administration 30,31. Furin protein could convert proBDNF to mBDNF, which not only promoted the survival of nerve cells and the growth of axons and dendrites by activating mBNDF/TrkB pathway but also reduced nerve cell apoptosis by inhibiting proBDNF/P75NTR pathway. In some studies, it has been found that MMP activation can lead to increased BDNF release and TrkB activation in cortical neuron cultures 32,33. The increase of intracellular zinc levels can increase MMP activity and BDNF expression 34. In the future study, the relationship between BDNF and Furin (or MMP) needs to be clarified in the pathological process of RIRI, which could be helpful in delaying the apoptosis of optic nerve cells as much as possible and promoting the survival of retinal nerve cells, and provide more beneficial treatment method for protecting the visual function of RIRI patients.

In this study, RNA sequencing was used to discover differently expressed genes in RIRI and to further explore the genes and enriched pathways that were reversed by DHF administration. We found a lot of differently expressed genes have been discovered to be involved in RIRI. For example, Gfap and Casp3 were up-regulated after RIRI. KEGG analysis also showed that the NF-kappa B signaling pathway, Jak-STAT signaling pathway, Toll-like receptor signaling pathway, PI3K-Akt signaling pathway, TNF signaling pathway, MAPK signaling pathway, Calcium signaling pathway, cAMP signaling pathway, and Apoptosis were significantly enriched. These findings were in agreement with previous studies 12,35-41. Different expression profile between RIRI and DHF+RIRI revealed reversed pathways responsible for DHF treatment. Several reversed pathways were overlapped with DHF induced BDNF/TrkB signaling, such as PI3K-Akt signaling pathway and Ras signaling pathway. And the other reversed pathways were indirect effects with the RIRI improvement and were also found in other studies, such as Toll-like receptor signaling pathway and NF-kappa B signaling pathway 40,41.

In summary, the present study revealed that systemic administration of DHF activates the BDNF/TrkB/NF-kB pathway to alleviate apoptosis and inflammation of RIRI to the retina and ganglion cells. Moreover, the reactivation of astrocyte was inhibited and mBDNF/proBDNF ratio was increased after DHF administration which led to decreased levels of apoptosis and increased levels of nerve cell survival. Therefore, the DHF treatment could be used as a potential therapeutic drug for ischemic ophthalmopathy in clinical practice.

## Supplementary Material

Supplementary figure S1 and table S3.

Supplementary table S1.

Supplementary table S2.
