# Supplementary material for: 7,8-Dihydroxyflavone alleviates apoptosis and inflammation induced by retinal ischemia-reperfusion injury via activating TrkB/Akt/NF-kB signaling pathway
Source: Int J Med Sci. 2022 Jan 1;19(1):13–24. doi: 10.7150/ijms.65733 (PMC8692126; doi:10.7150/ijms.65733)
Supplement: Supplementary file 1 — Supplementary figure S1 and table S3. [file ijmsv19p0013s1.pdf]

Supplemental files：

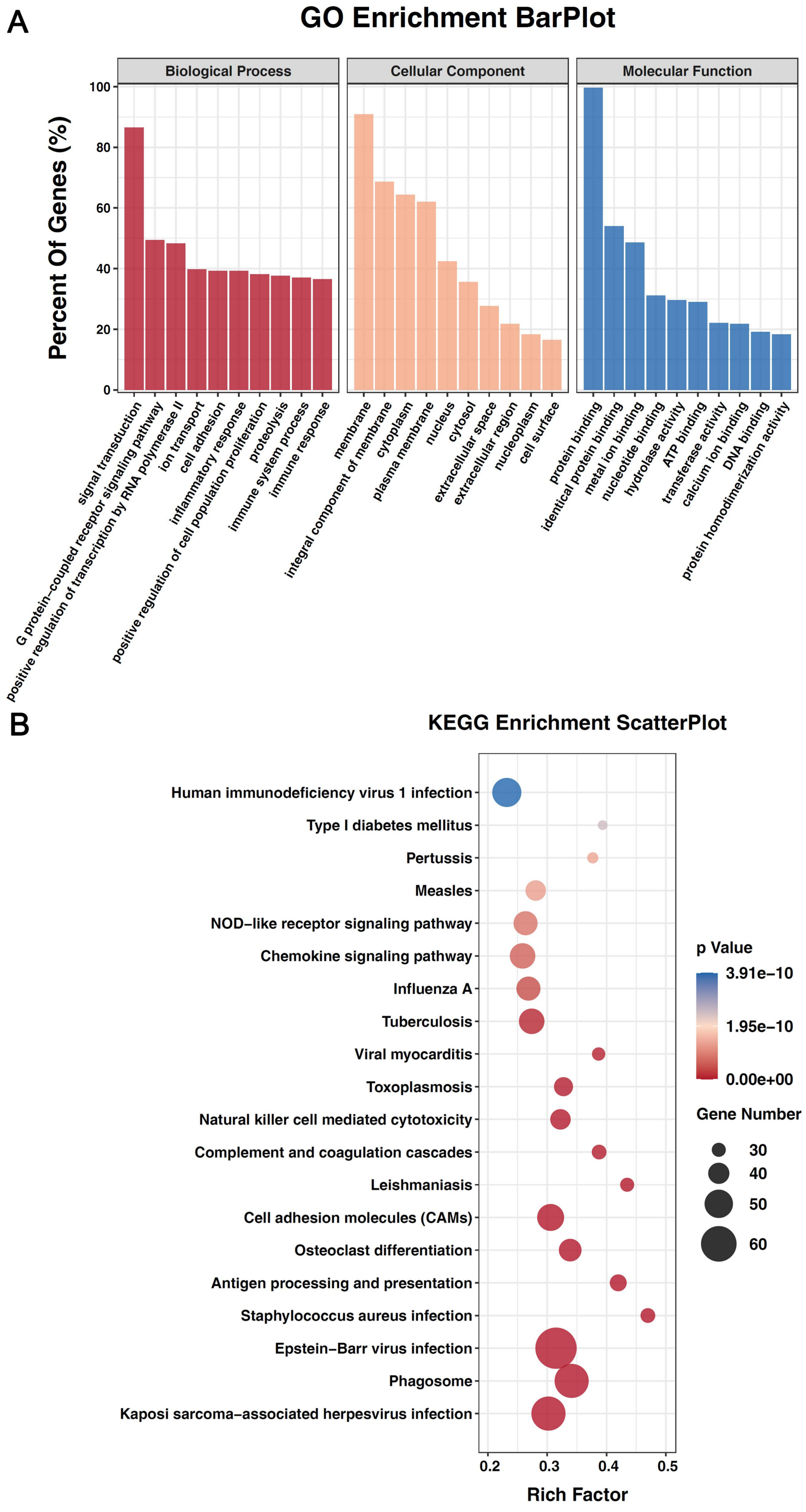

Figure S1. GO and KEGG enrichment analysis for DEGs in RIRI vs Sham.

Table S3 Primers designed for qRT-PCR

gene name	primer type	Primer Sequence (5'-3')
Casp1	Forward	GGCATGCCGTGGAGAGAAAC
	Reverse	GGTGTTGAAGAGCAGAAAGCA
Bcl2a1	Forward	CAGAATGGAGGCTGGGAAGAT
	Reverse	ACCATTCTCAAGGGAGCCAG
Ccl20	Forward	CAGCCAGTCAGAAGCAGCAAG
	Reverse	CGGCCATCTGTGTTGTGAAAC
Il17ra	Forward	GCTAGGAAAGCCCGTCGT
	Reverse	CTAGAGCCGGGAAGTCGAG
Pycard	Forward	AGCTGCAAAAGATTATGGAAGAGTC
	Reverse	GCCTGGTACTGTCCTTCAGTC
Rac2	Forward	GATGAGGCAATCCGAGCAGT
	Reverse	AGACTGGGAAGACACCCCTA
Tgfb1	Forward	GACCGCAACAACGCAATCTA
	Reverse	CGTGTTGCTCCACAGTTGAC
Myc	Forward	CTTACAATCTGCGAGCCAGGA
	Reverse	ATCGTCGTGACTGTCGGGTTT
Lgals3	Forward	GAGGAGCACTAACCAGGAAAATG
	Reverse	TCGAGGGTTTGGGTTTCCAG
Nefm	Forward	GAGATCGCCGCATATAGGAAA
	Reverse	GTGTACAGAGGCCCAGTGAT
Nova1	Forward	AGCACCAAGAGGACCAACAC
	Reverse	ACCCTCTCAGTAGTACCTGGAT
Pak1	Forward	AGCGAGCGCAGAAGTAGC
	Reverse	GGTGTTTCTCATCGGAGGGG
Gfap	Forward	GGGCGAAGAAAACCGCATC
	Reverse	ATTTGGTGTCCAGGCTGGTT
TNF-α	Forward	ACTGAACTTCGGGGTGATTG
	Reverse	GCTTGGTGGTTTGCTACGAC
IL-6	Forward	GGCTAAGGACCAAGACCATCCAA
	Reverse	TCTGACCACAGTGAGGAATGTCCA
IL-1β	Forward	CTCCATGAGCTTTGTACAAGG
	Reverse	GGGGTTGACCATGTAGTCGT
IFN-γ	Forward	AAGACAACCAGGCCATCAGCAA
	Reverse	GAACTTGGCGATGCTCATGAATGC
GAPDH	Forward	GCAAGTTCAACGGCACAG
	Reverse	GCCAGTAGACTCCACGACAT
